# Determining anatomical and electrophysiological detail requirements for computational ventricular models of porcine myocardial infarction

**DOI:** 10.1016/j.compbiomed.2021.105061

**Published:** 2022-02

**Authors:** Caroline Mendonca Costa, Philip Gemmell, Mark K. Elliott, John Whitaker, Fernando O. Campos, Marina Strocchi, Aurel Neic, Karli Gillette, Edward Vigmond, Gernot Plank, Reza Razavi, Mark O'Neill, Christopher A. Rinaldi, Martin J. Bishop

**Affiliations:** a Department of Biomedical Engineering, School of Biomedical Engineering & Imaging Sciences, King's College London, UK; b NumeriCor GmbH, Graz, Austria; c Gottfried Schatz Research Center, Biophysics, Medical University of Graz, Austria; d Institut de Rythmologie et de modélisation cardiaque (LIRYC), University of Bordeaux, France; e Medical University of Graz, Austria and BioTechMed, Graz, Austria; f Department of Cardiology, Guy’s and St Thomas’ Hospital, London, UK

**Keywords:** Patient-specific models, Cardiac electrophysiology, Myocardial infarction, Ventricular tachycardia

## Abstract

**Background:**

Computational models of the heart built from cardiac MRI and electrophysiology (EP) data have shown promise for predicting the risk of and ablation targets for myocardial infarction (MI) related ventricular tachycardia (VT), as well as to predict paced activation sequences in heart failure patients. However, most recent studies have relied on low resolution imaging data and little or no EP personalisation, which may affect the accuracy of model-based predictions.

**Objective:**

To investigate the impact of model anatomy, MI scar morphology, and EP personalisation strategies on paced activation sequences and VT inducibility to determine the level of detail required to make accurate model-based predictions.

**Methods:**

Imaging and EP data were acquired from a cohort of six pigs with experimentally induced MI. Computational models of ventricular anatomy, incorporating MI scar, were constructed including bi-ventricular or left ventricular (LV) only anatomy, and MI scar morphology with varying detail. Tissue conductivities and action potential duration (APD) were fitted to 12-lead ECG data using the QRS duration and the QT interval, respectively, in addition to corresponding literature parameters. Paced activation sequences and VT induction were simulated. Simulated paced activation and VT inducibility were compared between models and against experimental data.

**Results:**

Simulations predict that the level of model anatomical detail has little effect on simulated paced activation, with all model predictions comparing closely with invasive EP measurements. However, detailed scar morphology from high-resolution images, bi-ventricular anatomy, and personalized tissue conductivities are required to predict experimental VT outcome.

**Conclusion:**

This study provides clear guidance for model generation based on clinical data. While a representing high level of anatomical and scar detail will require high-resolution image acquisition, EP personalisation based on 12-lead ECG can be readily incorporated into modelling pipelines, as such data is widely available.

## Highlights

• Detailed scar morphology is required to simulate infarct-related arrhythmia.
• Representing both ventricles improves accuracy of arrhythmia simulations.
• Detailed anatomy and scar morphology are not required to simulate paced activation.
• Conduction velocities and tissue conductivities can be estimated from ECG data.
• ECG-based parameters improve accuracy of pacing and arrhythmia simulations.

## 1 Introduction

Patient-specific computational cardiac models have the potential to improve diagnosis and treatment of a variety of cardiac pathologies [1]. Recently, personalized models that combine cardiac anatomy and electrophysiology (EP) have been successfully applied to assess the risk of ventricular tachycardia (VT) in myocardial infarction (MI) [2], to optimize Cardiac Resynchronization Therapy (CRT) [[3], [4], [5], [6]], and to predict VT ablation targets [7]. While these models represent a step forward in the use of computational models in a clinical setting, the necessary level of anatomical detail and EP personalisation required to make accurate patient-specific predictions remains unclear, holding-back widespread clinical adoption.

To date, most patient-specific models applied to guide clinical decision making have relied on low-resolution cardiac magnetic resonance imaging (MRI) to derive patient anatomy [2,5,6]. Clinical MRI is typically acquired with reasonable in-plane resolution (2 × 2 mm) but low interslice resolution (6–20 mm). While such resolutions are adequate to reconstruct gross ventricular anatomy, this is not the case for regions of MI and fibrosis, often having complex morphology and thin (<8 mm [8]) anatomical isthmuses that are key substrates for sustaining VT circuits. While it is possible to reconstruct scar morphology between MRI slices through interpolation methods [9], this may introduce important errors in the faithful reconstruction of the narrow critical isthmus pathways, resulting in potential critical circuits being artificially closed-up, or unrealistic pathways being artificially created within the model. Subsequent simulations with the models may then result in important discrepancies in simulated activation sequences and VT morphologies, consequently reducing accuracy of model predictions of activation sequences, ablation targets or VT inducibility risk.

The impact of image resolution on scar morphology and VT circuits has been studied using image-based porcine models [10]. While the authors highlighted general agreement between VT circuits simulated on low-versus high-resolution image-based models, important differences in circuit location and dynamics were also highlighted, which could affect model-based prediction of patient-specific ablation targets.

Aside from anatomy, EP personalisation, has largely been absent, with the majority of modelling studies still relying on ‘average’ EP parameters derived from the literature [2,5,7]. This is largely due to the fact that robust identification of model parameters from EP clinical data remains a substantial challenge. Recent studies [11,12] have used intracardiac electrograms (EGMs) to compute local activation times (LATs) and activation-recovery intervals (ARIs) (a surrogate for action potential duration (APD)) to fit tissue conductivities and ionic conductances. However, invasive EP data is typically not readily available, and its use is often not aligned with the potential utility of models as non-invasive pre-procedural planning tools. Non-invasive EP data, such as ECG, are often available, but parameterization methods based on such data are more challenging and, consequently, limited to specific cases [13]. A recent study used the QRS duration on 12-lead ECG from heart failure patients to iteratively fit global Eikonal CV and to determine the site of latest ventricular activation as the optimal location for the left ventricular (LV) lead during CRT [3]. Another recent study used the wall thickness computed from cardiac computerized tomography (CT) to fit local Eikonal CV and simulate VT to predict ablation targets [14].

The impact of EP parameters on VT simulations remains unclear. EP model parameters, such as tissue conductivities and ionic current conductances, determine CV, excitability and refractoriness, which are important factors for VT generation [15]. Particularly, monomorphic VT is critically determined by wavelength, which is in turn determined by CV, APD, and tissue excitability. If the wavelength is shorter than the length of an anatomical circuit (most often determined by the scar substrate), re-entry can occur. Thus, adequate EP model parameterization, in combination with accurate representation of the anatomical MI substrate from imaging, may be crucial to investigate and/or predict VT inducibility using patient-specific models.

In summary, the majority of clinical EP studies use computational models based on images with lower (clinical) resolution and without personalisation of EP properties. In this study, we sought to investigate, and quantify, the functional implications of these modelling choices in a variety of EP scenarios, compared to models based on higher resolution images which more faithfully capture ventricular and scar anatomy, as well as the effects of patient-specific EP properties. To do this, we used high-resolution MRI and comprehensive invasive and non-invasive EP data obtained from a cohort of six pigs with induced chronic MI to build individualized models of ventricular anatomy and scar morphology, and to estimate EP parameters. These models were used to investigate the impact ventricular anatomy, scar morphology, and literature-based *versus* individualized CV had on paced activation sequences, and on VT inducibility and subsequent dynamics.

## 2 Methods

In this section, we begin by describing the details of the pre-clinical data acquisition (4.1), both anatomical (cardiac MRI), as well as functional (invasive electroanatomical mapping and non-invasive ECG recording) during a variety of pacing protocols. The process of creating computational finite element models with different levels of anatomical detail from the imaging data are then described (4.2), constituting the processes of segmentation and mesh generation, along with the techniques for interpolating scar information from the images to the models to create models with different levels of anatomical detail (i.e., different ‘resolutions’ of scar data). Methodological strategies for model functional personalisation, using the non-invasive pre-clinical EP measurements, are then described, including details of the important aspect of pre-processing of the EP data (4.3) and the pacing protocols and tuning strategies to fit model conductivity parameters (4.4). Section 4.5 then describes the complex VT-induction protocols used, with 4.6 detailing the data analysis procedures and how model findings were then compared to the invasive (‘ground truth’) pre-clinical mapping data and VT induction outcomes.

### 2.1 Experimental data

**Fig. 1:**
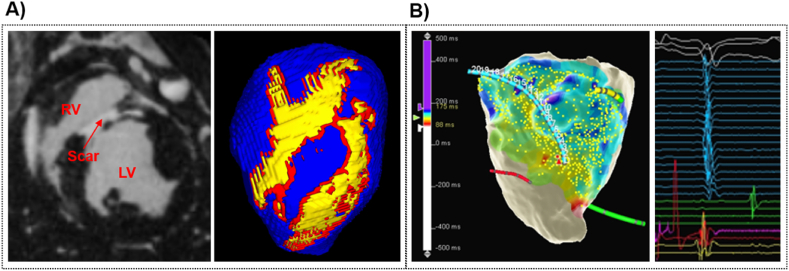
Experimental data. A) Example of LGE-MRI, showing the LV, RV, and the scar, with the corresponing LV (blue) segmentation, including scar (yellow) and border zone (red). B) Example of EP data acquired in-vivo using Precision™. Showing LAT map on the LV cavity computed by Precision™, and examples of acquired intra-cardiac EGMs (right).

All experimental protocols were approved by local and national institutional animal care and ethics committees. Six pigs underwent balloon-occlusion of the left anterior descending (LAD) coronary artery to create an ischemia-reperfusion MI model [16]. *In-vivo* late-gadolinium enhanced (LGE) MRI was performed on each pig a median of 53.5 days post MI. Images were acquired at an isotropic spatial resolution of 1 mm. Image segmentation was performed in Seg3D [17] by a single observer. LV myocardium was manually segmented in each image series. Scar was segmented according to the signal intensity range within the segmented myocardium, where the threshold for scar was ≤60% of the maximum intensity within the image, and the threshold for border zone (BZ) was ≤40% and >60% [18]. An example of LGE-MRI showing the right ventricle (RV), MI scar, and the LV with corresponding LV and scar segmentation is shown in Fig. 1-A).

EP study was performed following a median of 63 days post-MI using a Precision™ electro-anatomical mapping (EAM) system (EAMS, Abbott, Minnesota, MN, USA) with pacing stimulator. Intra-cardiac EGMs were recorded within the LV cavity using a multipolar mapping catheter (HD Grid™ or LiveWire™ duo-deca (Abbott, Chicago, IL)), during continuous pacing from the RV apex at a 500 ms. Programmed electrical stimulation (PES) was performed with an 8-beat drive train followed by up to four extra-stimuli to assess for VT inducibility. PES was repeated with isoproterenol infusion in the event that VT was not initially induced. VT was successfully induced in all 6 pigs and monomorphic VT that did not self-terminate within 30s was classified as sustained VT. Standard 12-lead ECGs were recorded throughout the EP study. Fig. 1-B) shows an example of an EAM recording, including LATs on the LV cavity and examples of intra-cardiac EGMs.

### 2.2 Anatomy modelling

#### 2.2.1 Image segmentation and post-processing

The RV blood pool was semi-automatically segmented in Seg3D [17] by a single observer using signal intensity thresholding and a connected component filter, followed by manual correction. The RV wall was created by dilating the RV blood pool uniformly by 4 mm [19]. The RV segmentation was combined with the LV, scar, and BZ segmentation.

To improve segmentation quality and remove any potential ‘stair-casing’ at outer surfaces, the bi-ventricular (BIV) segmentations, including scar and BZ, were up-sampled and smoothed using custom written software [20].

**Fig. 2:**
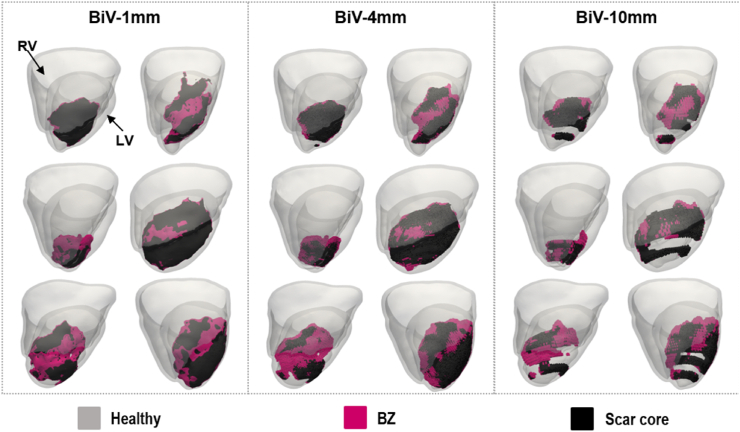
BIV anatomical models (viewed from the RV lateral wall) showing the RV and LV anatomies in grey, the scar core in black and BZ in pink. Models are shown for the six pigs and the three scar resolutions (1, 4, and 10 mm).

The scar segmentations were down-sampled in Seg3D in the *z*-direction from 1 mm to 4 or 10 mm to represent typical clinical LGE-MRI slice thickness. The down-sampled scars were reconstructed in 3D using a statistical shape approach based on the logOdds function [9], used in many previous clinical modelling studies [2,5,6]. Briefly, this involved application of Gaussian blurring and 3-dimenstional cubic interpolation, followed by logistic filtering to segment the interpolated and smoothed scar shape. For each dataset, this then created three ‘equivalent’ models (shown in Fig. 2): high-resolution scar (made from original 1 mm imaging data), and low-resolution scar (made from 4 mm or 10 mm down-sampled and reconstructed imaging data).

#### 2.2.2 Mesh and fibre generation

BIV meshes were generated from the high-resolution segmentations for each pig using custom written software based on the freely available library C-Gal [21]. Average mean edge length was approximately 325 μm, in line with other ventricular modelling studies [2,7]. BIV fibres were generated using a rule-based method [22]. An LV version of each BIV model was generated by extracting the LV sub-mesh using MESHTOOL [23]. Additional versions of each BIV and LV model were generated by replacing the high-resolution scar (1 mm) with the low-resolution scar (4 mm or 10 mm, Section 4.2.1). In total, 4 anatomical models were generated for each pig, namely BIV models for each scar resolution (1, 4, and 10 mm) and an LV model with high-resolution scar (1 mm), as shown in Fig. 1. We consider the BIV model with 1 mm scar resolution (BIV-1mm) as the *gold-standard* model for each pig.

### 2.3 Electrophysiology data processing

#### 2.3.1 Local EP properties

**Fig. 3:**
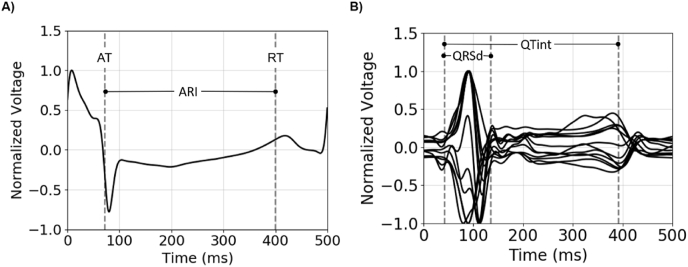
EP data properties. A) Example of EGM trace and computed AT, RT, and ARI. B) Example of 12-lead ECG and computed QRSd and QTint.

Activation and repolarization times (AT, RT respectively) and ARI were estimated from EGM data exported from Precision™ using bespoke algorithms implemented in OpenEP [24], based on conventional approaches applied to similar datasets [25]. Specifically, AT was defined as the point of maximum negative derivative (dV/dt) of the EGM (using the AT computed by Precision™ (EAMS, Abbott, Minnesota, MN, USA) to estimate an initial search window). This method of maximum negative gradient proved more consistent than a threshold-based approach for AT. RT was calculated using the Wyatt method as the maximum dV/dt prior to the T-wave peak, with a pacing-dependent search window after the AT used to constrain the EGM T-wave. If no peak of EGM was detected within the search window, a negative T-wave was assumed, and the minimum dV/dt prior to the T-wave peak was used. ARI was defined as the difference between AT and RT. Finally, the first and second ARIs recorded for each EGM were compared to ensure consistent contact in the recording process; if they differed by more than 10%, points were excluded from further analysis. An example of EGM trace and corresponding AT, RT, and ARI is shown in Fig. 3-A).

#### 2.3.2 Global EP properties

The first paced beat of the 12-lead ECG was excluded, and the subsequent 3 beats were averaged to obtain a single average ECG beat. The average ECG beat was converted to a vectorcardiogram (VCG), which was then used to define the start of the QRS complex as the point at which the spatial velocity of the VCG exceeded a threshold value [26]. To compute the QT interval (QTint), the end of the T-wave was calculated by finding the final peak of the ECG derivative during a paced beat, and extrapolating from the point of maximum gradient the return to baseline of the ECG (itself defined as the median value of the ECG 30 ms prior to the QRS) [27]. The median value of all ECG leads was used to exclude outliers, defined as leads with a QT longer than the median QT plus one standard deviation [27]. This process was repeated until no outliers were left and the final median was used to define the global QT-interval. An example of 12-lead ECG and corresponding QRSd and QT interval is shown in Fig. 3-B).

The mean APD of each pig was estimated from the QTint on the 12-lead ECG. Specifically, considering that the end of the T-wave corresponds to the total ventricular RT and that the QRS corresponds to the total ventricular AT, the QTint minus the QRSd provides an estimate of the mean APD within the ventricles.

Table 1 shows the QRSd, the QTint, QTint minus QRSd on 12-lead ECG, and the mean ARI on EGM computed for each pig using data obtained during RV pacing at a 500 ms cycle length.

**Table 1:** EP metrics computed on the 12-lead ECG and EGM data for each pig during RV pacing at a 500 ms cycle length. Showing QRSd, QTint, QTint-QRSd, and mean ARI. Values are quoted as mean ± standard deviation.

Pig	QRSd (ms)	QTint (ms)	QTint – QRSd (ms)	Mean ARI (ms)
1	92.52	391.53	299.01	317.13 ± 14.24
2	111.22	436.01	324.79	321.66 ± 20.04
3	104.33	440.42	336.09	275.64 ± 18.11
4	112.69	429.37	316.67	298.63 ± 30.14
5	106.29	406.13	299.83	331.45 ± 17.15
6	79.72	421.54	341.82	283.89 ± 31.63

### 2.4 Functional model EP personalisation

#### 2.4.1 EP setup

Baseline CVs were setup prior to fitting model parameters to the experimental EP data, with different CVs assigned to different regions of the heart. Transversely isotropic CV was assigned to healthy ventricular tissue, with baseline velocities of 0.67 and 0.3 m/s [28] in the longitudinal and transverse fibre directions, respectively. Reduced isotropic CV was assigned to the BZ, with a baseline CV of 0.15 m/s, based on experimental values [29], as used in previous studies [5,6]. The scar core was modelled as non-conducting.

A fast endocardial conduction (FEC) layer was added to the anatomical models using the universal ventricular coordinate (UVC) system [30], as done in previous work by our group [3,6]. Briefly, a layer was defined within the entire endocardial surface of both ventricles [3], including over scar and BZ using the transmural coordinate of the UVC, where a homogeneous thickness corresponding to 5% of the UVC transmural coordinate. The FEC over the RV side of the septum was kept in the LV-only models.

CVs within the FEC in the longitudinal direction was set to 6x the healthy [3] longitudinal CV (6 × 0.67 m/s at baseline), with the transverse CV equal to the healthy value (0.3 m/s) [3]. Since part of the FEC resides on top of scar, the CV within this region was set as 6x faster than the BZ CV (6 × 0.15 m/s at baseline). Such a representation was based on histological data showing a thin layer of surviving endocardial tissue over scar, where fibrosis and fibre disarray have been reported [29].

A His-Purkinje system (HPS) was generated for one of the models using custom written software [31] and the simulated activation sequences were compared against the same model with FEC. Details can be found in Supplemental Section 1.

#### 2.4.2 Simulating activation and 12-lead ECG

We estimated pacing locations using the UVC system to define the mid-septal point on the RV side, closest to the RV apex, which was known to be the target location for pacing in these experiments. Explicit experimental pacing locations were also obtained from the EP mapping system and simulation results for both estimated and experimental pacing locations were compared (see Supplemental Section 2 for details).

Ventricular activation sequences were simulated using the Eikonal equation, with activation starting from the designated pacing locations. The ATs from the Eikonal simulation were fed to the Reaction-Eikonal model [32] to obtain a transmembrane voltage, allowing subsequent computation of ECGs. Simulations were performed using the Cardiac Arrhythmia Research Package (CARP) [33].

Since the torso geometry of the pigs and the respective ECG electrode locations were not recorded in the original study, electrode locations were estimated by registering the standard 12-lead electrode locations from a human torso model, as done previously [26]. The distribution of transmembrane voltages simulated with the Reaction-Eikonal model were used to recover the extracellular potential at the registered electrode locations using the Poisson equation, as described previously [32]. Standard 12-lead ECG signals were computed from the extracellular potentials at the electrode locations.

To compare simulation results, the total activation time (TACT) was computed as the latest activation time at the ventricular base, and the QRSd was computed using the spatial velocity of the VCG, as described in Section 4.3.2.

#### 2.4.3 Tuning eikonal CVs

Activation sequences induced by RV pacing were simulated for each anatomical model using the Eikonal equation with the longitudinal CV within healthy tissue varying between 0.36 and 0.96 m/s in 0.01 m/s steps. CVs in the transverse direction of healthy tissue, within the BZ, and within the FEC layer were assigned using fixed ratios relative to the longitudinal CV of healthy tissue, namely, × 0.45, × 0.225, and × 6 [3], respectively. Thus, only one parameter was fit, speeding-up the parameterization process. The longitudinal CV was fit to either the TACT or the QRSd of the simulated ECGs:

***TACT-fit CV -*** In the first parameterization approach, the TACT at the base of the ventricle(s) was computed for each simulated activation sequence as a surrogate for QRSd. We chose the TACT at the base instead of the whole ventricle to avoid fitting the model to areas of severely-delayed activation within the scar. To fit the CV to the TACT, the difference between the TACT and QRSd of the corresponding experimental ECG was computed. The activation sequence, and corresponding CV value, whose TACT best matched the experimental QRSd was chosen [3], referred to as *TACT-fit CV.*

***QRSd-fit CV -*** Subsequently, a second step was introduced in which 12-lead ECGs were computed for activation sequences with the *TACT-fit CV* ±0.2 m/s in 0.01 m/s increments, and the QRSd of the simulated ECGs calculated. Similar to the previous approach, the activation sequence, and corresponding CV value, whose QRSd best matched the experimental QRSd was chosen, referred to as *QRSd-fit CV.* A schematic of the CV tuning process is shown in Supplemental Fig. S3.

### 2.5 VT induction simulations

#### 2.5.1 Action potential models

***Baseline AP model -*** The APD of the ten Tusscher model [34] for human ventricular cells was modified to represent porcine EP by multiplying the conductance of the slow and rapid rectifying Potassium currents, gKs and gKr, by a factor of 2.3, yielding an APD of 205 ms at a basic cycle length of 500 ms [35]. This was an important step due to the absence of a robust porcine-specific cell model and the need to compare directly with porcine pre-clinical EP data. The resulting model was also modified within the BZ to obtain a longer APD and reduced excitability, as done in the Virtual-heart Arrhythmia Risk Predictor (VARP) studies [2,7]. Specifically, the conductances of the peak sodium current, INa, the L-type calcium current, ICaL, and the rapid and slow rectifying Potassium currents IKr and IKs were reduced by 62%, 69%, 70 and 80%, respectively, within the BZ. This is referred to as *baseline AP model.*

***QT-fit AP model -*** The mean APD was estimated for each pig based on the experimental QRSd and QT-interval derived from the 12-lead ECG, as shown in Table 1. Using this metric, the APD of the baseline AP model was fitted for each pig by adjusting a multiplying factor for gKs and gKr in the ionic model. Similar to the CV tuning procedure, this was done by simulating the AP for a range of gKs and gKr values and selecting those where the resulting APD best fitted the APD estimate. The resulting model was also modified within the BZ as done in the *baseline AP model* and is referred to as *QT-fit AP model.*

#### 2.5.2 Tissue conductivities

Two sets of monodomain conductivities were derived using an automated approach [36], designed to tune required conductivities to desired CVs, taking into account model mesh resolution, EP parameters, and simulation setup. Tissue conductivities were tuned separately for healthy myocardium and BZ tissue, with the scar being defined as necrotic (zero conductivity), as in other works [2,[5], [6], [7], [8],^10^].

***VARP conductivities -*** To match the values reported in the VARP studies [2,7], healthy conductivities were computed using the baseline CVs for healthy tissue (0.67 m/s and 0.3 m/s) leading to longitudinal and transverse monodomain conductivities of 0.2262 S/m and 0.0642 S/m, respectively. In addition, the longitudinal BZ conductivity was set to be the same as the transverse healthy conductivity (0.0642 S/m) and the transverse BZ conductivity to 10% of this value (0.00642 S/m).

***QRSd-fit conductivities -*** A second set of conductivities were obtained that match the *QRSd-fit CVs* (Section 4.4.3) obtained for each pig, where the BZ conductivities were slow and isotropic, as in the baseline EP setup (4.4.1). The QRSd-fit CV and corresponding monodomain conductivities are listed in Supplemental Table S1.

#### 2.5.3 VT induction protocol

To investigate the impact of ventricular anatomy, scar morphology, and EP parameters on VT induction simulations, an S1–S2 protocol similar to the VARP protocol [2] was implemented. Specifically, 19 pacing locations were defined on each model using the UVC system (17 in LV, 2 in RV). To induce VT, 8 S1 stimuli were applied to each 19 (BIV) or 17 (LV) locations at a 600 ms basic cycle length. These were followed by a single S2 stimulus 250 ms (or APD + 45 ms for the *QT-fit AP* model) after the last S1. The stimuli were applied within a 2.5 mm radius for 1 ms with a stimulus strength of 100 μA/cm^3^. The S1–S2 interval was chosen based on a set of initial 2D simulations to define optimal stimulus prematurity. Propagation was simulated for 2s following the S2 stimulus to allow the induced VT to stabilize.

VT induction was simulated using the *VARP* conductivities, the *baseline AP model*, and the BIV-1mm, BIV-4mm, and BIV-10mm, and the LV-1mm models to investigate the impact of scar morphology (depending on imaging resolution) and ventricular anatomy on VT induction. In addition, one set of simulations using the BIV-1mm models, the *baseline AP model*, and the *QRSd-fit conductivities* were run to investigate the impact of individualized tissue conductivities on VT induction. Finally, another set of simulations using the BIV-1mm models, the *QRSd-fit conductivities*, and the *QT-fit AP model* was run to investigate the impact of individualized APD on VT induction.

### 2.6 Data analysis

The simulated activation sequences induced by RV pacing were compared against the experimentally measured LATs. To do so, the point clouds obtained from EAM were exported from Precision™ and rigidly registered onto the LV endocardium of the BIV-1MM model for each pig. The LV endocardial points of the anatomical model were compared with points in the EAM point clouds using a nearest neighbour method. The point-wise absolute difference between experimental and simulated LATs was computed and mean and standard deviations calculated.

In the VT induction simulations, VTs that did not self-terminate within the 2s following the S2 stimulus were classified as sustained [7]. The presence of sustained VT and its cycle length were determined automatically by computing the 12-lead ECG (Section 4.4.2) for the duration of the 2s of propagation following the S2 stimulus and analysing the signal morphology. Details are shown in Supplement Section 5. The outcome of VT simulations, namely positive (induced) or negative (non-induced) was compared against the experimental VT outcome.

## 3 Results

### 3.1 Impact of anatomy and parameterization strategy on conduction velocities

**Fig. 4:**
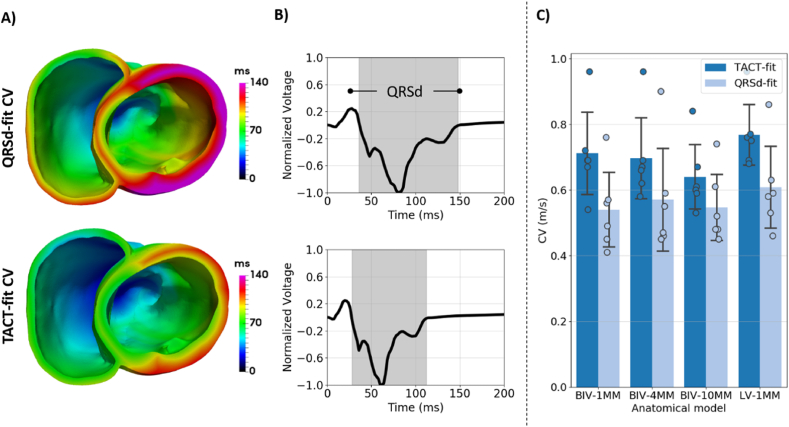
CV fitted to the TACT (TACT-fit CV) and the QRSd on 12-lead ECG (QRSd-fit CV). A) Examples of activation sequences with TACT-fit CV and QRSd-fit CV. B) Corresponding trace of lead V1 of the 12-lead ECG and the computed QRSd. C) CVs obtained using both parameterization methods, with bars and error bars corresponding to mean and standard deviation of CV values across all pigs, respectively, and filled circles indicating each the CV of each pig.

Fig. 4-A shows examples of activation sequences simulated using the BIV-1mm model, comparing the *TACT-fit* CV and *QRSd-fit* CV parameterization methods for one of the pigs. Here, activation is slower with the *QRSd-fit* CV than with the *TACT-fit* CV method, which is consistent with a longer QRSd for *QRSd-fit* CV than for *TACT-fit* CV, as shown in Fig. 4-B. Fig. 4-C confirms that the *QRSd-fit* CV is, on average, slower than *TACT-fit* CV for all anatomical models (0.57 ± 0.13 m/s *versus* 0.7 ± 0.12 m/s). In addition, *TACT-fit* predicts slower CVs with lower scar resolution (1 mm: 0.71 ± 0.14 m/s; 4 mm: 0.7 ± 0.13 m/s; 10 mm: 0.64 ± 0.11 m/s), showing a change of less than 10%, whereas *QRSd-fit CV* varies only slightly. Finally, both fitted CVs are slower in the BIV than the LV models for both *TACT-fit* (0.71 ± 0.14 m/s *versus* 0.73 ± 0.10 m/s) and *QRSd-fit* (0.54 ± 0.12 m/s *versus* 0.61 ± 0.13 m/s).

Since activation sequences also depend on pacing location, activation sequences were simulated using estimated and experimental pacing locations (Section 4.4.2) and the QRSd and TACT compared. The simulations show that both pacing locations yield very similar activation sequences (Supplemental Section 2).

The presence of a HPS may also affect activation sequences, particularly in pigs where it penetrates the ventricular wall deeply. Thus, activation sequences were simulated with models including a HPS system similar to humans and one similar to pigs and were compared against a model with a FEC layer (Section 4.4.1). The simulations show that activation sequences with a HPS and a FEC layer are also very similar (Supplemental Section 1).

### 3.2 Comparing simulated and experimental activation sequences

**Fig. 5:**
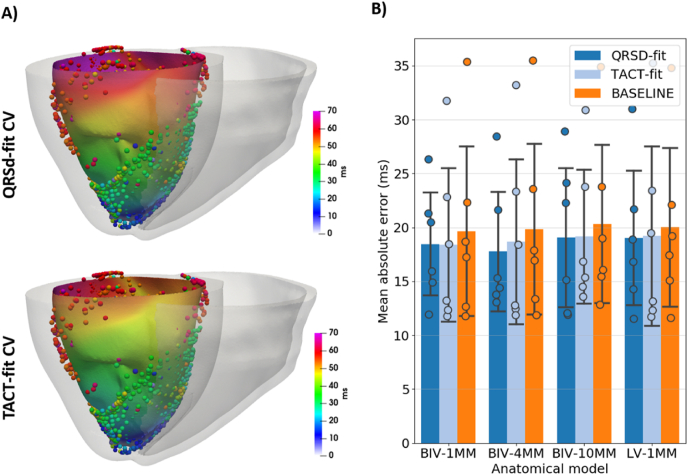
Comparison between experimental activation sequences and the baseline (constituting no modelling personalisation) and fitted sequences. A) Examples of simulated activation sequences on the endocardial surface for QRSd-fit and TACT-fit CVs. The overlapping spheres represent the measured LAT. B) Mean absolute error between experimental and simulated paced activation. Bars and error bars represent the mean and standard deviation over all pigs, whereas the mean error for each pig is shown as filled circles. Results are shown for the baseline CV (0.67 m/s), QRSd-fit CV, and TACT-fit CV.

The paced activation sequences simulated using the *baseline* CV (0.67 m/s), the *TACT-fit* CV, and the *QRSd-fit* CV were compared against experimental LAT from EAM. Fig. 5-A) shows examples of simulated activation sequences using the *QRSd-fit* CV and the *TACT-fit* CV on the endocardium, with the experimental LATs represented by the spheres. The examples show that while there is good agreement between the experimental LAT and both simulated sequences, the simulated LATs using the *QRSd-fit* CV match the experimental LATs more closely than *TACT-fit* CV, while local differences between experimental and simulated activation appear larger near the LV base. Fig. 5-B shows mean absolute errors over all pigs for each anatomical model and for *QRSd-fit*, *TACT-fit*, and *baseline* CVs (note that baseline corresponds to *no* model fitting/EP personalisation). While mean errors vary only slightly, the *QRSd-fit* CV yields slightly smaller errors than *TACT-fit* and *baseline* CV across models. The different scar resolutions have little impact on the mean absolute error, although a scar resolution of 10 mm yields slightly larger error for *QRSd-fit* CV compared to the BIV-1mm model, with mean errors of 19.05 (11.89–28.9) *versus* 18.46 (11.91–26.31). The presence of the RV slightly reduces the errors for *QRSd-fit* and *TACT-fit* CV, but has little impact on the error for *baseline* CV, with BIV-1mm and LV-1mm mean absolute errors of 18.46 (11.91–26.31) *versus* 19.02 (11.52–30.98) for *QRSd-fit* CV and 18.38 (11.76–31.73) *versus* 19.21 (11.70–35.24) for *TACT-fit* CV.

### 3.3 Impact of anatomy and EP parameters on VT inducibility and cycle length

**Fig. 6:**
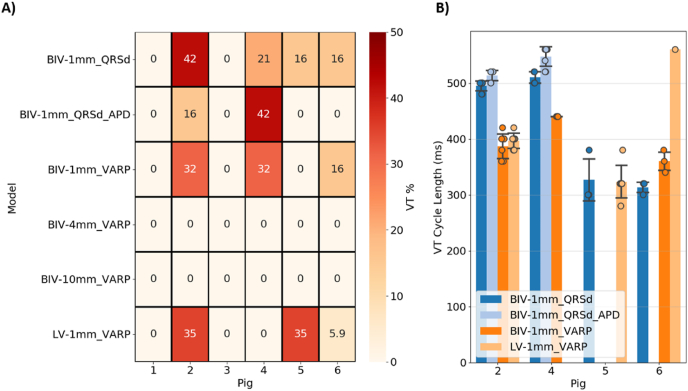
VT induction simulations: A) Heatmap of the percentage of VTs induced relative to the number of stimulus sites (19 for BIV, 17 for LV) for each model. B) Cycle lengths of the induced VTs for each model and pig, showing the computed cycle length for each pig as filled circles and mean and standard deviation over all pigs. “QRSd” and “VARP” correspond to QRSd-fit and VARP conductivities, respectively, and “APD” corresponds to the APD-fit action potential model.

A virtual VT induction protocol was applied to all anatomical models using the *VARP* and *QRSd-fit conductivities*, and the *baseline* and *QT-fit AP* models (Section 4.5). Fig. 6-A) shows a heatmap of the VTs induced in each model as the percentage of VTs relative to the number of stimuli locations (19 for BIV, 17 for LV). The cycle length of induced VTs was computed and the results are shown in Fig. 6-B).

#### 3.3.1 Scar morphology

Fig. 6-A) shows that no VTs were induced in any of the models with low-resolution scars (BIV-4mm and BIV-10mm) and *VARP conductivities*, whereas VT was induced in three pigs with high-resolution scars (BIV/LV-1mm) and *VARP conductivities*. In simulations using the *VARP conductivities*, VT induction mainly depended on the presence of an anatomical isthmus with functionally remodelled tissue (BZ) that can sustain re-entry. As shown in Fig. 2, none of the models with low-resolution scars contains an isthmus, as the LogOdds method was unable to reconstruct these from the more sparse MRI. Interestingly, no VTs were induced in Pigs 1 and 3 even with the highest resolution scars, which is in agreement with the absence of an anatomical isthmus in these models, as shown in Fig. 2. An example of VT generated in Pig 2 using the BIV-1mm model, but not with the BIV-4mm model, is shown Supplemental Section 6.

#### 3.3.2 Ventricular anatomy

Fig. 6-A) also shows that the presence of the RV plays an inconsistent role on VT inducibility. This is more evident when focusing on the simulations using the *VARP conductivities* for the BIV-1mm and LV-1mm models. Specifically, a similar percentage of VTs was induced in Pig 2 with the BIV-1mm and LV-1mm models (32% *versus* 35%), but a smaller percentage of VT was induced in Pig 6 with the LV-1mm model. Importantly, no VTs were induced in Pig 4 with LV-1mm model, but VT was induced in 32% of simulations with the BIV-1mm model. Conversely, no VTs were induced in Pig 5 with the BIV-1mm model, whereas VT was induced in 35% of simulations with the LV-1mm model. Moreover, the VT cycle length was longer in Pig 6 for the LV-1mm than for the BIV-1mm model but was similar in Pig 2.

**Fig. 7:**
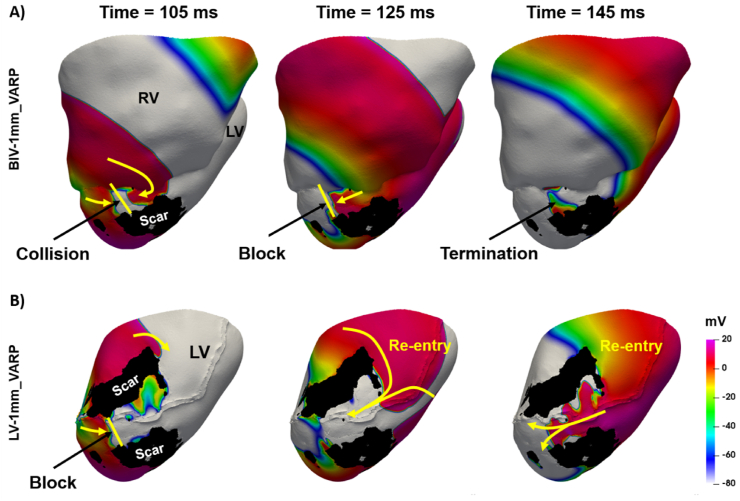
Example of ventricular tachycardia induction using a BIV-1MM (A) and a LV-1MM (B) model. The colors represent the transmembrane voltage. The yellow arrows indicate the direction of wavefront progragation and the yellow lines indicate propagation block. The scar is shown in black. The times are counted from the time of delivery the S2 stimulus.

Fig. 7 shows an example of where successful VT induction depends upon the use of a BiV or LV-only model. Specifically, Fig. 7-A shows (in Pig 5) unsuccessful VT induction where the BiV-1mm model is used, compared to successful VT induction in Fig. 7-B with the LV-1mm model. In the BIV-1mm model (Fig. 7-A), a wavefront coming from the RV collides with another coming from the LV apex (time = 105 ms), leading to conduction block (time = 125 ms) and wave termination (time = 145 ms). In the LV-1MM model (Fig. 7-B), propagation coming from the LV apex is blocked (time = 105 ms) at the mouth of the isthmus (between the two scar regions in black). Since no additional wavefront coming from the RV is present (due to the absence of the RV) to cause wavefront collision and termination (Fig. 7-A), the wavefront coming from the LV septum is able to enter the isthmus and re-enter (time = 125 and 145 ms).

#### 3.3.3 Tissue conductivity

Tissue conductivities (*QRSd-fit* or *VARP*) were also seen to affect VT inducibility, as seen in Fig. 6-A). Specifically, a higher percentage of VTs was induced in Pig 2 with *VARP conductivities* than with *QRSd conductivities* (42% *versus* 32%), but a smaller percentage was induced in Pig 4 (32% *versus* 21%). No VTs were induced in Pig 5 with *VARP conductivities*, but VTs were induced in 16% of the simulations with *QRSd-fit conductivities*. Interestingly, the same percentage of VTs were induced in Pig 6 for both conductivity sets. Moreover, the VT cycle length is generally longer in models with *QRSd-fit conductivities* than *VARP conductivities* (Fig. 6-B), which is in agreement with the slower CVs obtained with the *QRSd-fit* parameterization approach compared the baseline CV of 0.67 m/s (Fig. 4). Finally, when considering a binary outcome, *i.e.,* VT positive or negative, over all pacing locations, the *QRSd-fit conductivities* facilitate VT compared to *VARP conductivities*, as the former lead to 4 VT positive outcomes amongst the 6 pigs, compared with 3 VT positive outcomes amongst the 6 pigs for the later.

**Fig. 8:**
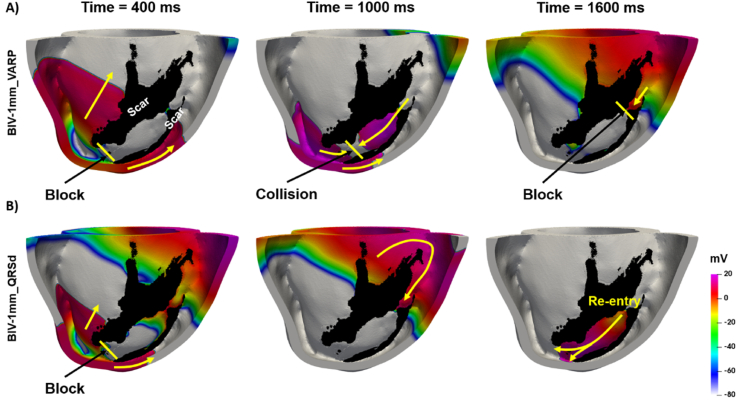
Example of ventricular tachycardia simulation using the A) VARP and B) QRSd-fit conductitivies. The colors represent the transmembrane voltage. The yellow arrows indicate the direction of wavefront progragation and the yellow lines indicate propagation block. The scar is shown in black. The times are counted from the time of delivery the S2 stimulus.

Fig. 8 shows an example of a scenario (in Pig 2) in which successful VT induction depends upon the tissue conductivities assigned to the model. For example, Fig. 8-B shows VT being successfully induced using the BIV-1mm model with *QRSd-fit conductivities*, whereas Fig. 8-A shows unsuccessful VT induction using the *VARP conductivities.* Using the *VARP conductivities*, which are higher than the *QRSd conductivities*, the wavefront propagates faster around the scar (time = 400 ms) and collides with another wave coming from near the RV apex (time = 1000 ms). The subsequent wave is then blocked at the isthmus entry (time = 1600 ms), since the tissue is not fully recovered, thus, leading to VT termination. Conversely, when using the *QRSd-fit conductivities*, the wave takes longer to travel around the scar (time = 400 ms), allowing sufficient time for tissue recovery at the anatomical isthmus (time = 1000 ms), thereby leading to re-entry (time = 1600 ms).

#### 3.3.4 APD

The APD of the *QT-fit AP* model is, on average, 115 ms longer (299–340 ms; Table 1) than the baseline (205 ms), but it is similar to the mean ARI computed on the intra-cardiac EGM of each pig (275–331 ms; Table 1). Fig. 6-A) shows that using the *QT-fit AP* model also affects VT inducibility. This is more evident when focusing on simulations using the *QRSd-fit conductivities* (BIV-1mm_QRSd and BIV-1mm_QRSd_APD)*.* Specifically, no VTs were induced in Pigs 5 and 6 with *QT-fit AP,* but VT was induced in 16% of simulations using the baseline model. A smaller percentage of VTs was induced in Pig 2 using the *QT-fit AP* model compared to the baseline model (16% *versus* 42%), whereas the opposite was observed in Pig 4 (42% *versus* 21%). Moreover, the VT cycle length was longer in the *QT-fit AP* models than the baseline model, consistent with the longer APD of the former. Finally, when considering a binary outcome, the *baseline AP* model facilitates VT compared to the *QT-fit AP* model, as the former lead to 4 out of 6 VT positive outcomes, compared with 2 VT positive outcomes amongst the 6 pigs for the later using the *QRSd-fit conductivities*. An example of VT induced using a BIV-1mm models with *QRSd-fit conductivities* and the baseline, but not with the *QT-fit* AP models is described in Supplemental Section 7.

## 4 Discussion

Patient-specific models are increasingly used to address clinical questions [5,6] and to predict therapy outcome [[2], [3], [4],^7^]. However, the level of anatomical, morphological, and EP detail needed to construct ventricular EP models of MI for use in making robust clinical predictions and guide personalized therapies remains unclear. In this study, we investigated the role of scar morphology, ventricular anatomy, and EP personalisation on paced activation sequences and on VT inducibility using porcine models of MI built from experimental imaging and EP data. Access to unique high-resolution pre-clinical MRI data, along with corresponding invasive (EAM) and non-invasive (ECG) recordings, made it possible to create like-for-like low/high resolution models, with/without EP personalisation, along with providing a ‘ground truth’ (EAM) to directly compare our model predictions with.

Our main finding is that the level of model anatomical detail (scar or ventricular) has little effect on the simulation of paced activation sequences but is more important when simulating VT induction; furthermore, EP model personalisation is important for both the simulation of pacing and VT-induction. More specifically, we showed that: 1) high image resolution (∼1 mm) is required to accurately represent scar morphology and conducting isthmuses, which directly effects simulations of VT induction and dynamics; 2) representing only the LV or both ventricles effects VT induction; 3) tuning CVs to the QRSd on 12-lead ECG improves model accuracy when compared to experimentally measured activation sequences; 4) tissue conductivities tuned to the QRSd render VT more inducible compared to values obtained from the literature and more closely match the experimental VT outcome. This provides evidence that a higher level of anatomical and morphological detail is required when constructing patient-specific models for simulating VT but not paced activation. Moreover, non-invasive EP personalisation improves model accuracy compared to experimental data.

### 4.1 The role of scar morphology and image resolution

Clinical MRI usually has low out-of-plane resolution, often varying from 6 to 20 mm [12]. This hinders accurate segmentation of infarct scars, especially thin structures, such as isthmus, which are a known critical substrate for VT. A shape reconstruction method based on the LogOdds function was recently developed to interpolate scar morphology between image slices and was shown to lead to accurate VT induction compared to scar morphology extracted from higher resolution images [9]. This technique has been used in several studies [2,7] to reconstruct scar morphology, including by our group to simulate activation/repolarization sequences [5,6]. In this study, scar morphology segmented from the high-resolution images (1 mm isotropic) and scar morphologies reconstructed from down-sampled images (4 and 10 mm slice thickness) using the LogOdds method [9] were included in our models. While the LogOdds method was able to reconstruct the bulk scar shape, isthmuses were not accurately reconstructed, being often entirely absent in lower resolution reconstructions (Fig. 2).

Re-entry requires an anatomical circuit that is longer than the activation wavelength [15]. Whilst models may be specifically parameterised to obtain the correct wavelength, the critical isthmus must still be accurately represented in a model, otherwise, VT cannot be induced. Furthermore, the presence or absence of small ‘extra’ pathways might also significantly affect the dynamics of the induced VT. Our results are at odds with a previous simulation study [10], as well as VT simulations using the LogOdds method [9], where the authors suggested that image resolution, and thus scar morphology, did not play a significant role in VT inducibility or dynamics. Here, we found that VT induction was not possible in any pig models (0/6) with lower resolution scar representations (4 mm or 10 mm), but VT was successfully induced in 3/6 models with ‘full’ resolution (1 mm) scar reconstructions (Fig. 6-A). Considering the VT positive outcome for all pigs during experimental PES and that VT was induced in 4 out of 6 models, our results suggest that accurately representing scar morphology is crucial for predicting VT using computational models.

Activation sequences during RV pacing are less affected by scar morphology than VT inducibility (Fig. 4), leading to similar errors compared to experimental data (Fig. 5-B). Thus, patient-specific models including scar morphology obtained from low-resolution images (BIV-4mm and BIV-10mm) can still accurately predict activation sequences during pacing and are, thus, appropriate for activation and repolarization simulation studies [3,5,6].

### 4.2 The role of ventricular anatomy

The LV is often regarded as more important than the RV for VT generation, since infarct scars are typically located within the LV myocardium, and LV disfunction is generally more detrimental to health. Thus, several computational studies model only the LV anatomy [5,14,37]. In this study, models of both BIV and LV-only anatomy were generated and the impact of ventricular anatomy on simulations of RV pacing and VT induction were investigated.

Pacing simulations predict that the using an LV-only model requires using larger CVs to fit the TACT or QRSd to the experimental QRSd compared to the BIV models, as shown in Fig. 4-C. During RV pacing, activation spreads quickly through the RV and then enters the LV, thus, acting as a current source for LV activation (Fig. 4). This decreases the TACT and QRSd in the BIV model, requiring slower CV to fit simulated activation sequences to experimental QRSd compared to the LV model. However, an LV-only model leads to only slightly larger errors relative to experimentally-measured activation compared with a BIV model (Fig. 5-A). This is likely due to the effect of the RV on activation being largely confined to the epicardial surface, whereas endocardial activation is mostly driven by the FEC layer. Importantly, when removing the RV from the anatomical model, we did not remove the FEC layer covering the RV side of the septum, which is likely to mitigate the lack of current sources from the RV wall. Thus, while the RV affects paced activation sequences, the effect is minor, which suggests that LV-only models are adequate for simulating paced activation.

VT simulations predict that the presence of the RV can both facilitate or hinder VT induction (Fig. 6-A, and Fig. 7), as well as influence the VT cycle length (Fig. 6-B). Specifically, one extra pig model became VT-inducible with the addition of the RV (Fig. 6-A), although another model also became VT non-inducible. Monomorphic VT is often determined by the presence and the morphological and functional properties of an anatomical isthmus [8,38]. Occlusion of the LAD coronary artery, as done in the pigs used in this study, leads to infarct scars covering the anterior side of the septum and parts of the anterior LV wall [16]. Thus, scar isthmuses might be located very close to the RV junction (Fig. 2). Consequently, the presence of the RV in computational models will be a determinant factor of VT inducibility and dynamics (Fig. 7), particularly relevant in the case of LAD occlusion and septal scar. Thus, it is important to include the RV in patient-specific simulations for both pacing and VT inducibility, as previously done [2,7], particularly when scars are located within the septum.

### 4.3 The role of EP parameterization

EP parameterization has been absent in most recent patient-specific models [2,[5], [6], [7]], which have relied on literature parameters. Invasive EP data is often not available, and its use to tune model parameters [11,14] is often incompatible with their use as non-invasive pre-procedure tools. Non-invasive EP data, such as 12-lead ECG is more widely available, but identifying EP parameters from such data is challenging [13].

A recent study fitted global Eikonal CV to the QRSd on clinical 12-lead ECG to determine the site of latest ventricular activation as the optimal location for the LV lead during Cardiac Resynchronization Therapy [3]. The authors used the simulated TACT as a surrogate for QRSd, which is comparable with our *TACT-fit* parameterization approach. The *TACT-fit* approach was shown to predict the optimal CRT electrode position accurately [3], as it does not affect the location of the latest activation site. However, the correspondingly-derived CV will affect the wavelength and, consequently, VT inducibility [15]. In this study, the approach was extended by computing the 12-lead ECG from simulated activation and fitting the QRSd instead of the TACT to the experimentally-acquired QRSd. RV pacing simulations predict that TACT is longer than the QRSd (Supplemental Fig. S2). Moreover, using TACT to tune CV lead to faster CV (Fig. 6-C), shorter QRSd (Fig. 4-B), and slightly larger errors relative to experimentally measured activation than the *QRSd-fit* approach (Fig. 5-B). Thus, the *TACT-fit* approach is likely inappropriate to obtain conductivities in the case of arrhythmia simulations and was not used for VT induction simulations in this study.

Local heterogeneity of EP characteristics, such as APD, ERP, and CV play a prominent role in the occurrence of uni-directional block and re-entry [15]. In MI, these heterogeneities are augmented within the scar, especially at the BZ [29,39]. Recent studies have modified ionic models to obtain a longer APD and reduced excitability, as well as reduced tissue conductivity with the BZ [2,7]. In this study, we implemented a similar approach (*VARP conductivities* and *baseline AP model* with modified BZ properties) and compared it against the personalized *QRSd-fit conductivities* and *QT-fit* AP models.

Each parameter set used in this study lead to mixed results in terms of the number of induced VTs (Fig. 6-A). However, when considering a binary outcome (VT positive or negative), the *QRSd conductivities* were pro-arrhythmia, facilitating overall VT induction compared to the *VARP conductivities*. Importantly, the *QRSd-fit conductivities* lead to a VT positive outcome in all models with an anatomical isthmus (4 out of 6), where VT is likely to be induced under arrhythmogenic EP conditions, such as slow CV. In fact, since VT was induced experimentally in all pigs, the BIV-1mm model with *QRSd-fit conductivities* and *baseline AP model* was the most accurate for VT outcome prediction. Conversely, simulations using the *QT-fit* AP model led to less VT positive outcomes (Fig. 6-A) compared to the *baseline AP model* and the experimental VT outcome. This is likely due to the longer APD in the QT-fit AP model compared to the baseline AP model, as short APD is generally more arrhythmogenic than long APD [15]. Moreover, the QTint minus QRSd may overestimate the APD compared to optical measurements [35], affecting restitution properties and consequently VT induction. Thus, CV/conductivity personalisation is important for representing both VT inducibility and paced activation compared to using values obtained from the literature. However, APD personalisation based on the QTint on 12-lead ECG is not beneficial for predicting VT outcome.

### 4.4 Clinical implications

The models created in this study were based solely on non-invasive data (MRI and 12-lead ECG) and showed good agreement with invasively acquired data (EAM and VT outcome). Our results clearly demonstrate the need for higher resolution imaging for model creation, particularly for VT simulations where accurate scar morphology representation is crucial. Moreover, the *QRSd-fit* parameterization approach based on 12-lead ECG data can be easily incorporated into modelling pipelines, since such data is typically widely available. Moving forward, computational models built from non-invasive clinical data can be used as complementary clinical tools for risk and therapy outcome predictions with few adjustments to clinical pipelines.

## 5 Limitations

The image resolution of the data used in this study to create the anatomical models was higher than typical clinical resolutions (1 mm *versus* 4–10 mm out-of-plane) but still insufficient to capture anatomical isthmuses in 2 of the 6 pigs, which hindered virtual VT induction. It is possible that VT was induced via different mechanisms in these pigs during the experimental EP study or that VT was facilitated by isoproterenol infusion. Unfortunately, EAM during VT was not available to our study to investigate additional mechanisms. The relatively short delay between MRI and EP data collection (∼10 days) was unavoidable, although, due to the relative maturity of the infarct at this stage, we do not believe any significant remodelling would have occurred in this time period.

Changes to ionic current conductances were applied within the BZ but not within the ventricles for the VT inducibility simulations (Section 4.5.1). However, it has been reported that no significant regional differences in APDs are present in pigs [35] and that such differences do not play a prominent role in VT induction [40].

Model mesh resolution (mean of 325um) was chosen to be similar to other clinical modelling studies [2,7]. Furthermore, the methodology used to tune tissue conductivities [36], in the personalisation approach presented, was performed based on this mesh resolution and other simulation parameters, to match the desired CV.

The LV extent of the FEC is unclear. Purkinje fibres arborizations are mostly located in the lower third of the ventricles [41,42], but endocardial breakthrough sites have also been identified near the base of the LV [43]. Our choice of a FEC layer extending over the full length of the endocardial surface is based on a previous simulation study [3], showing that tuning the CV (based on the QRSd on 12-lead ECG) of a FEC model covering only the lower third of the LV did not improve simulation errors relative to clinical measurements of LAT.

## 6 Conclusion

For the first time, this work has directly assessed the different functional EP consequences of using low-versus high-resolution MRI to construct ventricular MI models, and how the differences uncovered are augmented by model EP personalisation. This study provides clear guidance towards determining model requirements to make accurate predictions and to facilitate clinical translation of patient-specific ventricular models of MI. The simulation results presented here suggest that the RV and detailed scar morphology should be included in patient-specific models used to simulate VT, whereas these are likely not required for simulating paced activation. Moreover, personalizing CV/conductivities based on 12-lead ECG is beneficial for both pacing and VT simulations and is also easily incorporated into models using the *QRSd-fit* parameterization approach.

## Sources of funding

This work was primarily supported by a 10.13039/501100000274BHF Project Grant (PG/18/74/34077) and 10.13039/100010269Wellcome Trust Innovator Award (213342/Z/18/Z) to MJB. MJB also acknowledge support of King's Health Partners R&D Research Challenge Fund (Reference: MC_PC_18052) and MRC New Investigator Award (MR/N011007/1). This research was funded/supported by the 10.13039/501100000272National Institute for Health Research (10.13039/501100000272NIHR) 10.13039/100014461Biomedical Research Centre and CRF based at Guy's and St Thomas' NHS Foundation Trust and King's College London. The views expressed are those of the author(s) and not necessarily those of the NHS, the NIHR or the Department of Health. This work was also supported by the Wellcome EPSRC Centre for Medical Engineering at King's College London (WT 203148/Z/16/Z). GP acknowledges the support of the grants I2760–B30 from the Austrian Science Fund (FWF), BioTechMed-Graz Flagship award ILearnHeart, and MedalCare 18HLT07 from the EU.

## Declaration of competing interest

The authors declare that they have no known competing financial interests or personal relationships that could have appeared to influence the work reported in this paper.
