# Supplementary material for: Determining anatomical and electrophysiological detail requirements for computational ventricular models of porcine myocardial infarction
Source: Comput Biol Med. 2022 Feb;141:105061. doi: 10.1016/j.compbiomed.2021.105061 (PMC8819160; doi:10.1016/j.compbiomed.2021.105061)
Supplement: Multimedia component 1 [file mmc1.docx]

**Supplement**

1. **His-Purkinje system**

The presence of the His-Purkinje system (HPS) is crucial for synchronous ventricular activation. While pig and human hearts bare several similarities, the morphology of their HPS differs. Specifically, the Purkinje-Muscle Junctions (PMJ) in pigs are known to penetrate the ventricular wall, whereas these are located just below the endocardial surface in humans[1]. This leads to a much smaller transmural delay in ventricular activation and a shorter QRS duration (QRSd) in pigs than in humans[2].

While explicitly modelling the HPS has the advantage of simulating more realistic ventricular activation, generating the networks and simulating activation within the HPS is complex and requires specialized software. An alternative approach is to include a layer of fast FEC[3], as done in this study (see Section 2.4 in the main study). To quantify the impact of using a fast endocardial conduction (FEC) layer instead of explicitly modelling the His-Purkinje System (HPS), two HPS networks were generated for one of the BIV-1MM models using a method as presented in detail in Gillette et. al.[4]. In summary, a baseline network architecture was constructed by growing fascicles of the Purkinje network from specific root locations across the entirety of the endocardium (*human-like*) and connecting all fascicles through a His-bundle system. To address this difference in PMJ depth between humans and pigs, we created a second network (*pig-like*), where the penetration depth of the PMJs within the entire network was set to approximately 50% of the wall thickness (based on the transmural coordinate of the UVC system). Figure S1 shows the *human-like* (A) and *pig-like* (B) HPS networks.

The HPS was modelled as a branching network of one-dimensional cubic Hermite elements separated by discrete gap junctions, modelled as fixed resistances[5]. The DiFrancesco-Noble equations[6] were used to govern membrane electrical activity in the HPS and HPS propagation was governed by the monodomain equation[5]. The PMJs were connected to a minimum of 10 and a maximum of 20 myocardium elements, depending on local edge-length. PMJ resistance was set to 1000KΩ. Gap junctional conductivity in the HPS was tuned to yield a CV of 4.02m/s using an automatic parameterization approach[7].

While explicitly modelling the HPS has the advantage of simulating more realistic ventricular activation, generating the networks and simulating activation within the HPS is complex and requires specialized software. An alternative approach is to include a layer of fast endocardial conduction (FEC)[3], which yields fast ventricular activation to generate a synchronous heartbeat. To compare the effect of explicitly modelling the HPS against a FEC layer, we included a thin (0.5mm) FEC layer with 6x faster CV than the healthy CV longitudinal to fibres (6x0.67=4.02m/s), as done previously[8].

In both HPS and FEC models, myocardium conductivities were tuned to yield healthy CV longitudinal (0.67m/s) and transverse (0.3m/s) to fibres using the same automatic approach. In addition, no scar was included in the models, to avoid confounding our results with the effect of scar on local activation patterns.

Activation sequences and 12-lead ECGs were simulated during RV pacing (Section 2.5.1; main text) using both *human-like* and *pig-like* HPS networks. As we simulated RV pacing instead of sinus rhythm, HPS activation occurred via retrograde (from muscle to HPS) instead of anterograde (HPS to muscle) propagation.

The HPS simulations show that the relative error in QRSd between the endocardial and intramural PMJs is 8.98%. In addition, the difference in QRSd between intramural PMJ and a corresponding model with a FEC layer is 2.38%, whereas this difference is 12.5% for the endocardial PMJs. Thus, His-Purkinje networks were not included in the models used in the main study and a FEC layer was used instead.


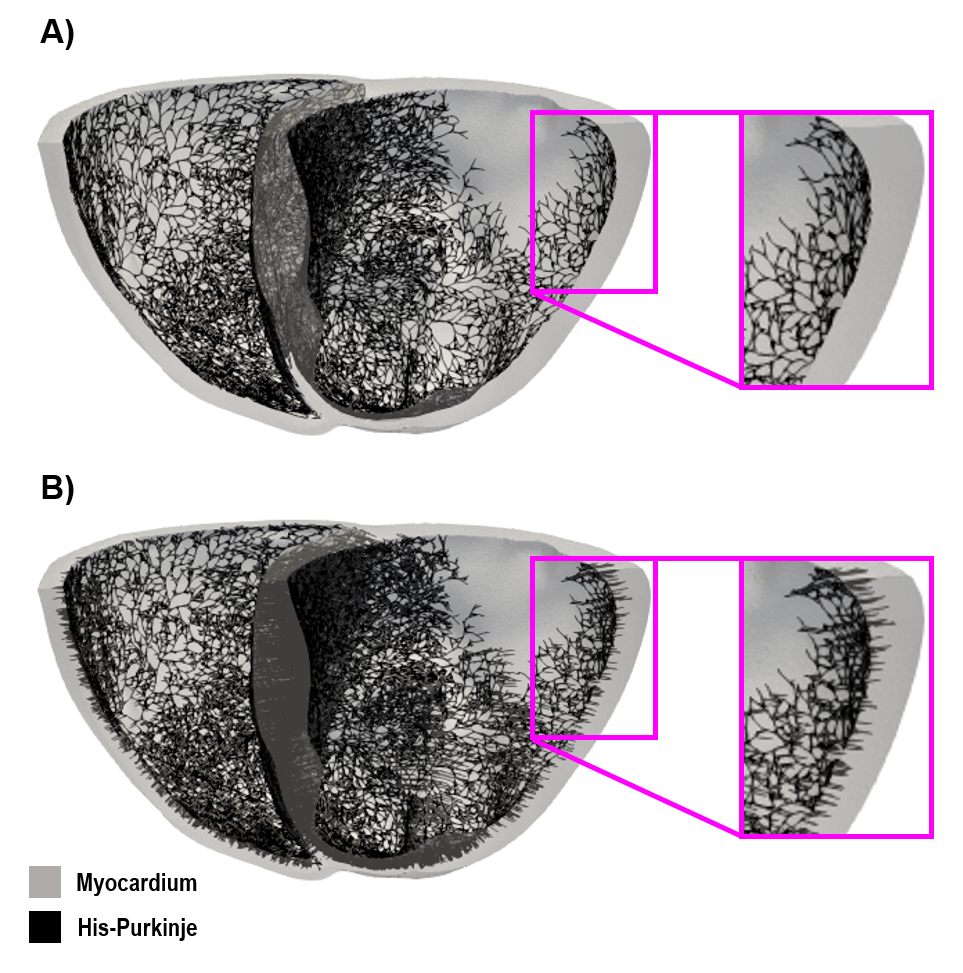


Figure S1: A) Human-like (endocardial) and B) pig-like (intramural) His-Purkinje networks within a bi-ventricular model without scar. The myocardium is shown in grey and the networks are shown in black. The highlighted regions (pink) show the penetration depth of the Purkinje-Muscle Junctions in the human-like and pig-like network.

1. **Estimated versus experimental pacing locations**

Reconstructing activation sequences requires selecting appropriate early activation sites (or pacing locations) and model parameters, such as tissue conductivities. Since pacing locations are not always available, it is important to develop and evaluate methods that can estimate pacing locations based on a-priori knowledge. Therefore, the TACT of activation sequences simulated using the pacing locations estimated using the UVC system (Section 4.5.1 in the main text) were compared against those simulated with the actual experimental pacing locations, which were exported from the EP mapping system for each pig. In addition, the 12-lead ECG and QRSd were computed for each simulated activation sequence.

Figure S2 shows similar activation sequences (Figure S2-A) and QRSd (Figure S2-B) for simulations using the estimated and experimentally-derived pacing locations. This is confirmed in Figure S2-C), which shows that for both pacing locations yield similar TACT (134.83±17.44ms *versus* 143.43±17.88ms) and QRSd (99.33±11.23ms *versus* 107.00 ±14.69ms). Figure S2-C also highlights that the TACT is always substantially larger than the QRSd.

This demonstrate that, when pacing locations are not precisely known, estimated locations can be used without substantially affecting parameterization results. Thus, the estimated pacing locations were used in all other simulations in the main study.

**
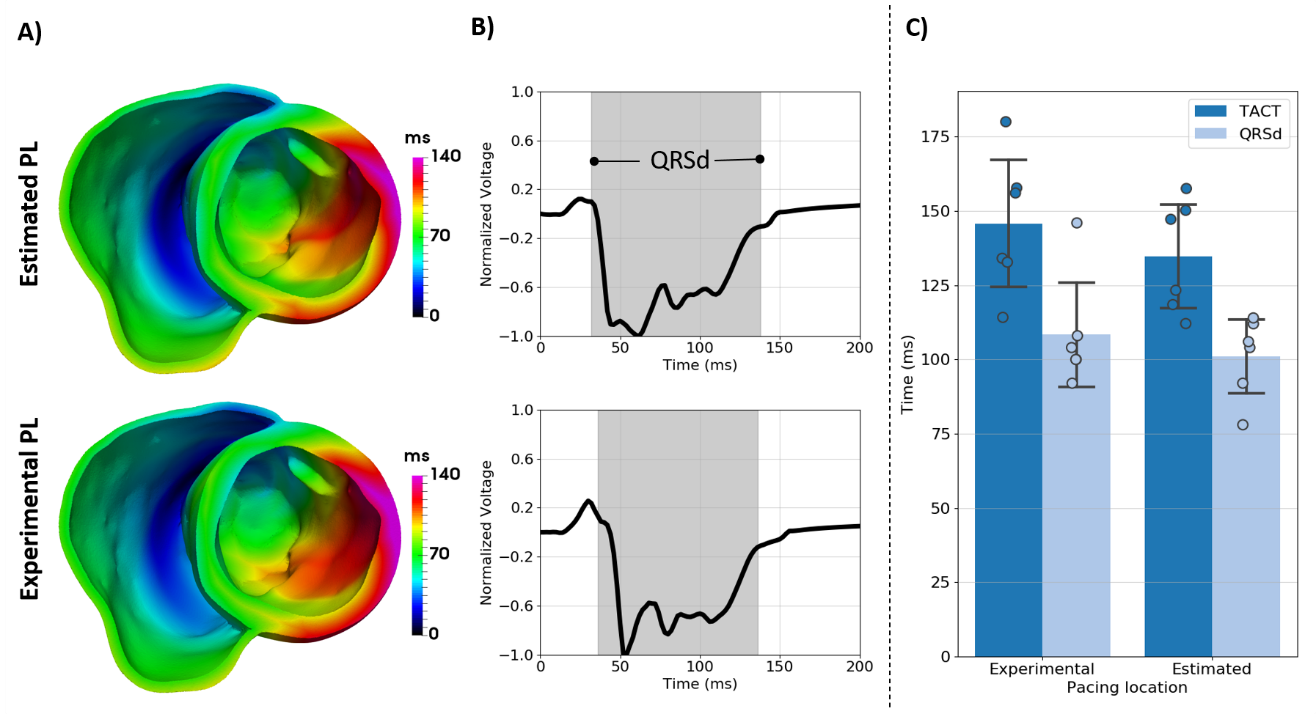
**

Figure S2: Experimental versus estimated pacing locations. A) Simulated activation sequences. B) QRS duration (QRSd) computed on 12-lead ECG (shown on lead V1). C) Filled circles show the total activation time (TACT) and QRSd for each of the 6 pigs; bars and error bars represent the mean and standard deviation of TACT and QRSd across all pigs, respectively.

1. **Tuning conduction velocities**

Figure S3 shows a schematic of the parameterization approach for tuning Eikonal conduction velocities (CV) using the total activation time (TACT-fit CV) and the QRS duration on 12-lead ECG (QRSd-fit CV).

*
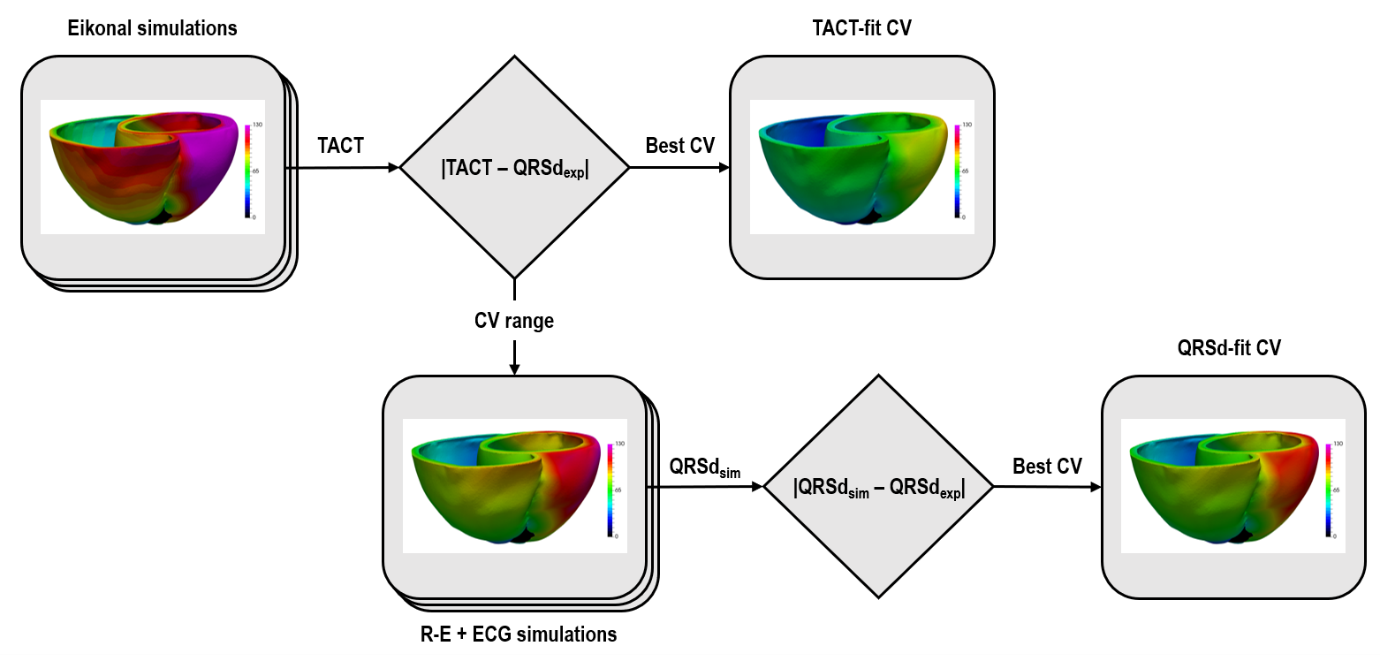
*

Figure S3: Tuning Eikonal conduction velocities (CVs). Eikonal simulations are run with varying CVs. The total activation time (TACT) is computed and compared to the experimental QRS duration (QRSd_exp_). The CV that leads to the smallest difference (best CV) is selected (TACT-fit CV). In addition, Reaction-Eikonal (R-E) and ECG simulations are run for a CV range centered around the best TACT-fit CV +/- 0.2 m/s. The simulated QRS duration (QRSd_sim_) is compared against the QRSdexp, and an updated CV obtained (QRSd-fit CV).

1. **QRSd-fit conductivities**

Table S1: QRSd-fit CV and respective monodomain conductivities for each pig. “lon”: longitudinal, “trans”: transverse, “BZ”: border zone, “Cond”: conductivity.

| Pig | CV_lon (m/s) | Cond_lon (S/m) | CV_trans (m/s) | Cond_trans (S/m) | CV_BZ (m/s) | Cond_BZ (S/m) |
| --- | --- | --- | --- | --- | --- | --- |
| 1 | 0.56 | 0.167398 | 0.252 | 0.050224 | 0.126 | 0.020772 |
| 2 | 0.41 | 0.102078 | 0.1845 | 0.032926 | 0.0922 | 0.015256 |
| 3 | 0.57 | 0.172244 | 0.2565 | 0.051534 | 0.12825 | 0.008557 |
| 4 | 0.45 | 0.117801 | 0.2025 | 0.032929 | 0.10125 | 0.01661 |
| 5 | 0.49 | 0.134854 | 0.2205 | 0.041761 | 0.11025 | 0.018096 |
| 6 | 0.76 | 0.281286 | 0.342 | 0.077752 | 0.171 | 0.029876 |

1. **Determining VT outcome and cycle length**

The presence of VT and VT cycle length were determined automatically using signal processing techniques on lead V1 of the 12-lead ECG. Specifically, 12-lead ECGs were computed (see Section 4.5.1 in the main text) for VT induction simulations for the 2s following the delivery of the S2 stimulus in the VT induction protocol. The signal in lead V1 was normalized and filtered using a third order Butterworth digital filter with a critical frequency of 0.25. The negative peaks on the normalized and filtered signal were computed, where the minimum height, distance, width, and prominence were set to 0.05, 6, 2, 0.1, respectively. The V1 signals were processed using the Python Scipy package. The amplitude of the last 200ms were computed and VT was defined as positive if this was above 0.025. If VT was positive, the interval between the last two peaks was computed as the VT cycle length. Figure S4 shows examples of lead V1 signals where VT is positive (Figure S4-A) and negative (Figure S4-B).


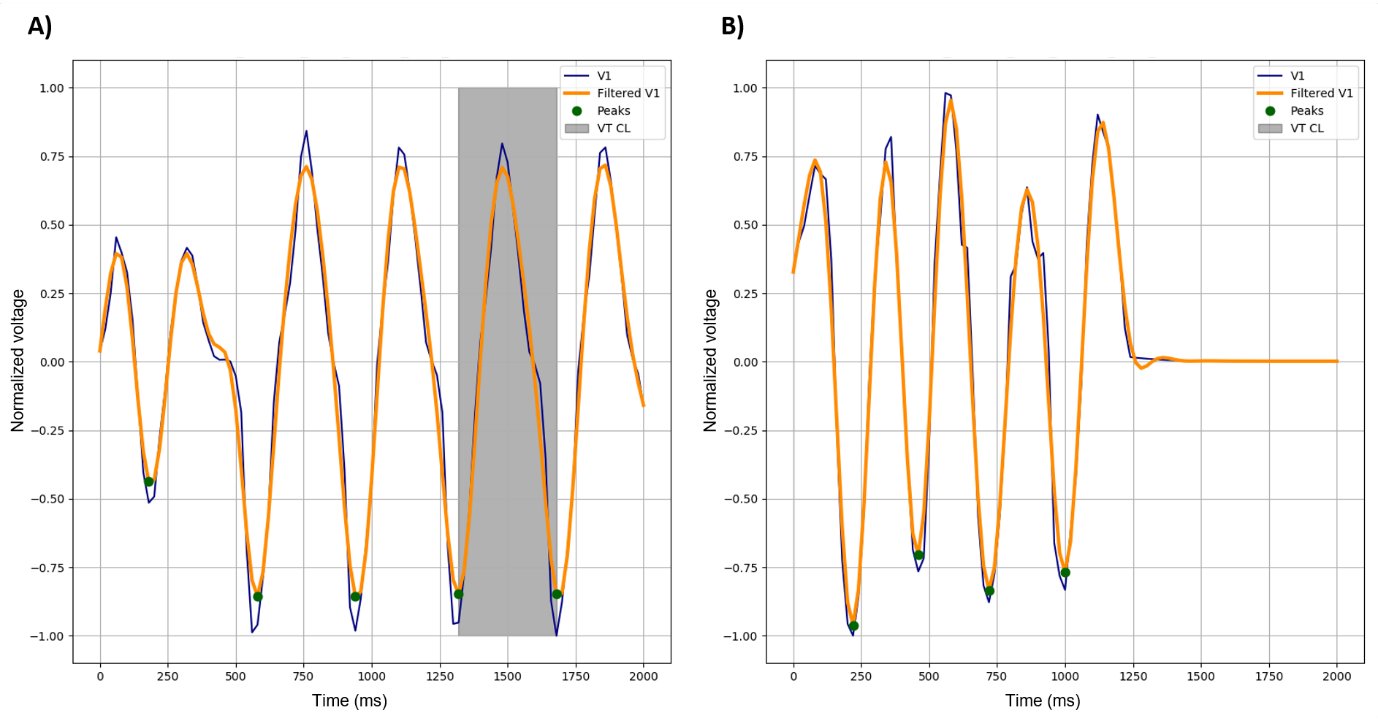


Figure S4: Example of automatic VT detection and VT cycle length computation. The original sginal on lead V1 of the 12-lead ECG is shown in blue, the filtered signal in orange, the computed peaks in green and the cycle length in grey. A) Positive VT oucome and cycle length. B) Negative VT outcome.

1. **Effect of scar morphology on VT**

Figure S5 shows an example of VT generated using the BIV-1mm model but not with the BIV-4mm model. The *VARP conductivities* were used in both cases. Figure S5-A) shows that when the scar morphology derived from the high-resolution images is included in the model (BIV-1mm), thin isthmuses are represented in the model. Importantly, when an isthmus is present, uni-directional functional block can occur at the mouth in one direction (time = 300ms), allowing the initiation of re-entry (time = 500ms). Conversely, when the scar morphology is derived from low-resolution images, thin isthmus cannot be represented at the model and may be replaced by dense scar, causing a region of anatomical block in both propagation directions (Figure S5-B). In this case, re-entry cannot be induced.


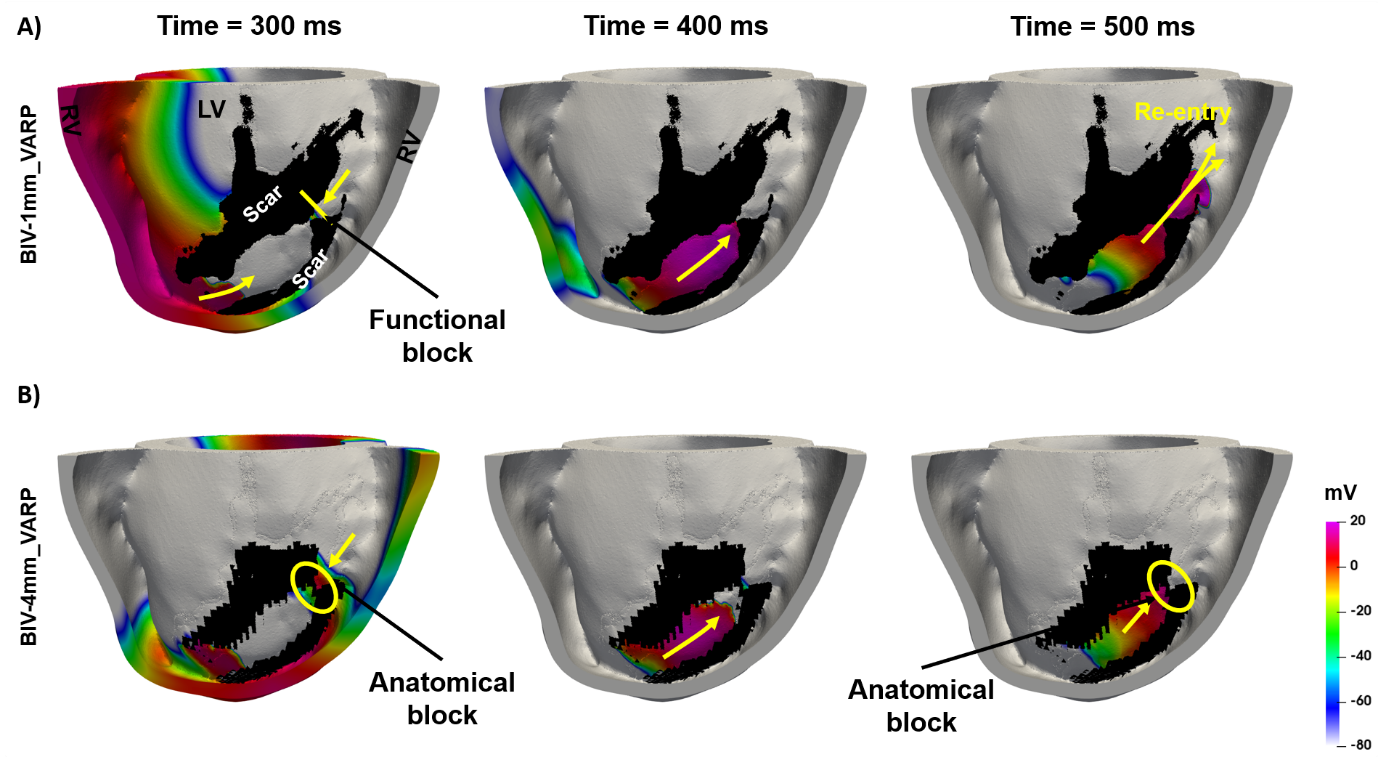


Figure S5: Example of ventricular tachycardia simulation the A) BIV-1mm and the B) BIV-4mm models with VARP conductivities. The colors represent the transmembrane voltage. The yellow arrows indicate the direction of wavefront progragation, the yellow line indicates functional propagation block, the yellow circles highlight a region of anatomical block. The scar is shown in black. The times are counted from the time of delivery the S2 stimulus.

1. **APD**

Figure S6 shows an example of VT induction using a BIV-1mm models with *QRSd-fit conductivities* and the *baseline* (Figure S6-A) and *QT-fit* AP (Figure S6-B) models. Notice that the propagation wave is shorter in the *baseline AP model* than in the *QT-fit* *model*. Here, three wavefronts propagate from the stimulus site (time = 350ms): one towards the isthmus, one towards the RV, and going around the scar. In Figure S6-A), the isthmus wavefront is blocked at the isthmus mouth, while the other two wavefronts continue to propagate (time = 500ms). Later, the wavefront coming from the RV is able to enter the isthmus and initiate re-entry (time = 650ms). Conversely, in Figure S6-B), the wavefront is not blocked at the isthmus mouth (time = 350ms), leading to wavefront collisions (time = 500 and 650ms) and eventually termination. It is worth noting that the time of S2 stimulus was adjusted to match the longer APD in the *QT-fit* model, so that the S2 wave arrives at the back of the S1 at approximately the same instant as in the baseline model. However, with the *QT-fit* model, the S2 wavefront is more depolarized and S1 wave tail is more repolarized than with the baseline model. This affects the effective refractory period only slightly, but it is enough to allow propagation instead of uni-directional block, which happens with the baseline model.

**
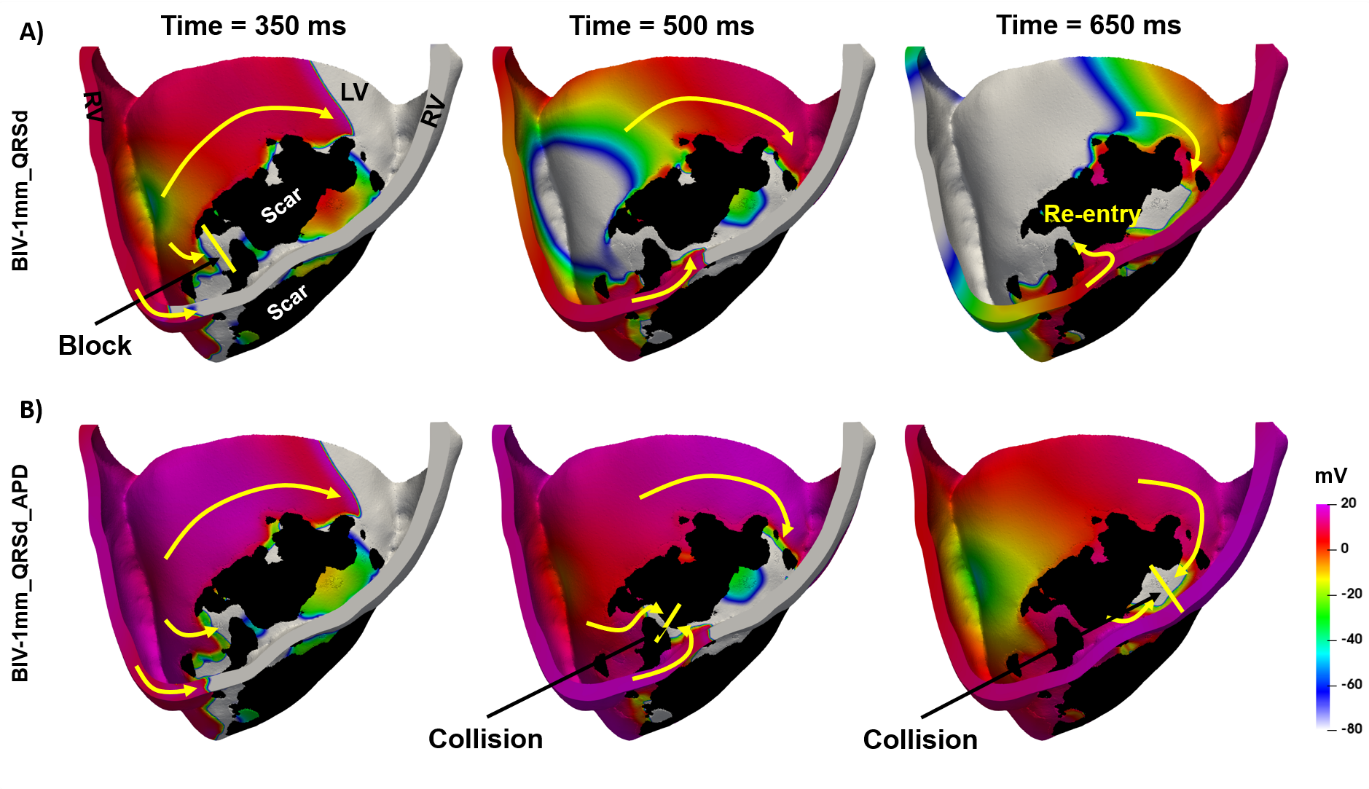
**

Figure S6: Example of ventricular tachycardia induction using a BIV-1mm model with the baseline (A) and QT-fit AP (B) models. The figures show a view from a cut plane at the RV lateral wall. The colors represent the transmembrane voltage. The yellow arrows indicate the direction of wavefront progragation and the yellow lines indicate propagation block. The scar is shown in black. The times are counted from the time of delivery the S2 stimulus.

**References**

[1] J. Tranum-Jensen, A. A. Wilde, J. T. Vermeulen, and M. J. Janse, “Morphology of electrophysiologically identified junctions between purkinje fibers and ventricular muscle in rabbit and pig hearts,” *Circ. Res.*, vol. 69, no. 2, p. 429, 1991, doi: 10.1161/01.RES.69.2.429.

[2] R. P. Holland and H. Brooks, “The QRS complex during myocardial ischemia. An experimental analysis in the porcine heart,” *J. Clin. Invest.*, vol. 57, no. 3, pp. 541–550, 1976, doi: 10.1172/JCI108309.

[3] E. R. Hyde *et al.*, “Beneficial Effect on Cardiac Resynchronization from Left Ventricular Endocardial Pacing Is Mediated by Early Access to High Conduction Velocity Tissue: Electrophysiological Simulation Study,” *Circ. Arrhythmia Electrophysiol.*, vol. 8, no. 5, pp. 1164–1172, 2015, doi: 10.1161/CIRCEP.115.002677.

[4] G. K *et al.*, “Automated Framework for the Inclusion of a His-Purkinje System in Cardiac Digital Twins of Ventricular Electrophysiology.,” *Ann. Biomed. Eng.*, p. To appear, 2021.

[5] P. M. Boyle, M. Deo, G. Plank, and E. J. Vigmond, “Purkinje-mediated effects in the response of quiescent ventricles to defibrillation shocks,” *Ann. Biomed. Eng.*, vol. 38, no. 2, pp. 456–468, 2010, doi: 10.1007/s10439-009-9829-4.

[6] D. DiFrancesco and D. Noble, “A Model of Cardiac Electrical Activity Incorporating Ionic Pumps and Concentration Changes.,” *Philos. Trans. R. Soc. London*, vol. 307, no. 1133, pp. 353–398, 1985.

[7] C. Mendonca Costa, E. Hoetzl, B. M. Rocha, A. J. Prassl, and G. Plank, “Automatic Parameterization Strategy for Cardiac Electrophysiology Simulations.,” *Comput. Cardiol. (2010).*, vol. 40, pp. 373–376, Oct. 2013, [Online]. Available: http://www.ncbi.nlm.nih.gov/pubmed/24729986.

[8] A. W. C. Lee *et al.*, “A rule-based method for predicting the electrical activation of the heart with cardiac resynchronization therapy from non-invasive clinical data,” *Med. Image Anal.*, vol. 57, pp. 197–213, Oct. 2019, doi: 10.1016/j.media.2019.06.017.
